# Characterization of Dried Blood Spot Quality Control Materials for Lysosomal Enzyme Activity Assays Using Digital Microfluidic Fluorometry to Detect Lysosomal Storage Disorders in Newborns

**DOI:** 10.3390/ijns11020044

**Published:** 2025-06-10

**Authors:** Paul Dantonio, Tracy Klug, Golriz Yazdanpanah, Christopher Haynes, Hui Zhou, Patrick Hopkins, Robert Vogt, Rachel Lee, Carla Cuthbert, Konstantinos Petritis

**Affiliations:** 1 Newborn Screening and Molecular Biology Branch, Division of Laboratory Sciences, National Center for Environmental Health, Centers for Disease Control and Prevention, Atlanta, GA 30341, USA; pbd1@cdc.gov (P.D.); czu1@cdc.gov (G.Y.); cph7@cdc.gov (C.H.); huw3@cdc.gov (H.Z.); rfv1@cdc.gov (R.V.); nmo1@cdc.gov (R.L.); ijz6@cdc.gov (C.C.); 2 Missouri State Public Health Laboratory, Newborn Screening Unit, 101 N. Chestnut Street, Jefferson City, MO 65101, USA; tracy.klug@health.mo.gov (T.K.); patrick.hopkins@health.mo.gov (P.H.)

**Keywords:** newborn bloodspot screening, lysosomal storage disorder specimen quality, dried blood spots

## Abstract

Newborn bloodspot screening for one or more lysosomal storage disorders (NBS-LSD) is currently performed by many public health NBS laboratories globally. The screening tests measure activities of selected lysosomal enzymes on dried blood spot (DBS) specimens collected from newborns by the heel stick method Because these assays measure enzyme activity, the quantitative results are dependent on the particular analytical method. DBS quality control (DBS QC) materials with assay-specific certified values that span the relevant range from typical to LSD-affected newborns are an important component of quality assurance in NBS laboratories. The Newborn Screening Quality Assurance Program (NSQAP) at the U.S. Centers for Disease Control and Prevention (CDC) provides public health NBS laboratories with DBS QC sets for NBS-LSD comprising four admixtures of pooled umbilical cord blood and a base pool made from leukodepleted peripheral blood and heat-inactivated serum. To evaluate the suitability of these materials for use with digital microfluidics fluorometry (DMF) assays which can currently measure the activity of four enzymes (acid α-galactosidase (GLA); acid β-glucocerebrosidase (GBA); acid α-glucosidase (GAA); and iduronidase (IDUA)), CDC collaborated with the Newborn Screening Unit at the Missouri State Public Health Laboratory (MSPHL). Using MSPHL criteria, we found that the certified results from each of two DBS QC lots collectively spanned the range from typical (screen negative) to enzyme deficient (screen positive) newborn DBS levels for each of the four lysosomal enzymes measured. The range included borderline results that would require repeat screening of the newborn under the MSPHL protocol. We conclude that these DBS QC preparations are suitable for use as external quality control materials for DMF assays used to detect LSDs in newborns.

## 1. Introduction

Dried Blood Spot Quality Control (DBS QC) materials are used routinely in newborn bloodspot screening (NBS) laboratories to ensure the accuracy of test results. The Newborn Screening Quality Assurance Program (NSQAP) at the U.S. Centers for Disease Control and Prevention (CDC) produces and distributes DBS QC materials for use as external reference materials by newborn screening laboratories [1,2,3]. NSQAP certifies mean and variance values for these DBS QC materials based on analytic methods in current use by NBS laboratories.

Lysosomal storage disorders (LSDs) include more than 50 enzyme deficiencies, several of which are candidates for detection by Newborn Bloodspot Screening (NBS) [4,5]. Four LSDs (Pompe disease, mucopolysaccharidosis I, mucopolysaccharidosis II, and Krabbe) have been added to the U.S. Recommended Uniform Screening Panel (RUSP) since its inception [6]. CDC produces and distributes DBS QC sets with certified mean and variance values for six lysosomal enzyme activities determined by MS/MS functional assays [7]. Activity values measured by digital microfluidic fluorometry (DMF) have also been certified for four lysosomal enzymes—acid α-galactosidase (GLA); acid β-glucocerebrosidase (GBA); acid α-glucosidase (GAA); and iduronidase (IDUA)—which are used for the detection of Fabry, Gaucher, Pompe, and mucopolysaccharidosis type I diseases, respectively.

The Missouri State Public Health Laboratory (MSPHL) newborn screening program has extensive experience using DMF for NBS-LSD [8], having screened about 854,166 newborns from 2013 to 2024. In a collaborative study, CDC analyzed two consecutive lots of DBS QC using the DMF method and applied the MSPHL criteria for categorizing the quantitative results on each of the four lysosomal enzymes tested. The distribution of categorical results for each DBS QC level provided an assessment of their relevance to the identification of affected newborns.

This paper also shows that agreement between the DMF instrument and tandem mass spectrometry (MS/MS) results is possible and while the DMF method and the MS/MS method show different values, harmonization could be a way to bring these values into closer agreement [9].

## 2. Materials and Methods

### 2.1. DBS QC

The DBS QC set with four different levels was prepared as previously described [7] using pooled umbilical cord blood (UCB) from either Carolinas Cord Blood Bank (lot 1708) or LifeSouth Community Blood Centers (lot 1808). Lot 1808 was prepared one year after lot 1708. The pooled UCB were adjusted to 50% hematocrit, and a base pool was made from leukodepleted whole blood (Tennessee Blood Services, Memphis, TN, USA) adjusted to 50% hematocrit with charcoal-stripped, delipidated serum (Seracare Life Sciences Inc., Milford, MA, USA) that had been heat-inactivated at 56 °C for 4 h. The two intermediate pools were made from admixtures of the base pool and the cord blood pool: one with 5% cord blood and 95% base pool (QC-Low) and the other with 50% of each pool (QC-Mid). The highest level DBS QC samples were spotted directly from the pooled cord blood (QC-High), and the lowest level were spotted directly from the base pool (QC-BP). Each of the four levels was applied at 100 µL per spot onto Whatman 903 filter paper DBS collection cards approved for use as a collection device, so that each card contained 15 DBS, all made from one of the four levels. The cards were allowed to dry overnight under ambient conditions and then packaged in low-permeability Ziplock bags containing desiccant packs. The packaged cards were stored at −20 °C until analyzed. For analysis, bagged cards were removed from the freezer and allowed to reach room temperature before removal from the desiccated bag.

Appropriate safety controls, including engineering measures, administrative policies and procedures, and personal protective equipment, were implemented for all procedures, based on a site-specific risk assessment that identified physical, health, and procedural hazards. All dried blood spot specimens used in this study were manufactured by the Centers for Disease Control and Prevention’s Newborn Screening Quality Assurance Program (NSQAP) using commercially available blood products. This activity was reviewed by CDC, deemed research not involving human subjects, and was conducted consistent with applicable federal law and CDC policy.

### 2.2. Study Protocol and Data Analysis

DBS were tested using the procedures established for NBS according to the product insert (Baebies Inc., Durham, NC, USA). Briefly, 3 mm punches from the DBS specimens were placed in 96-well microtiter plates, 100 µL of extraction buffer was added to each sample, and the plate was placed on an orbital shaker for 30 min at room temperature. Stop buffer, reagents, pre-wash extraction buffer, and calibrators were added to the testing cartridge. A 3.5 µL aliquot of each DBS extract was then added to the cartridge using the multichannel pipette specified in the protocol (Thermo-Fisher Finnpipette, Waltham, MA, USA, catalog number 46200300). Plates were analyzed on the SEEKER instrument immediately after preparation.

To obtain the certified values for the mean and standard deviation of the lysosomal enzyme activities, lot 1708 was analyzed more than 20 times over a two-week period within two months after preparation. It was again analyzed repeatedly 12 months later, concurrently with the newly prepared lot 1808. All data, including values below the specified limits of quantification, were transferred to a spreadsheet program (Excel, Microsoft) and analyzed using the statistical functions for mean and standard deviation. Data from 20 separate analytical runs, in which results on lot 1708 were within two standard deviations of the certified mean for all four enzymes on all four materials, were used to obtain certified values for lot 1808. Categorical results were assigned as high-risk, borderline, or within normal range, based on cutoff values used by MSPHL during the testing period.

## 3. Results

### 3.1. Quantitative Enzyme Activities

In both QC lots, GLA showed the highest activity and GBA the lowest (Table 1). The activity levels were similar in both lots except for GLA, which was nearly 40% higher in lot 1808. Variability between lots of each material was comparable: the composite coefficient of variation (CV) for results from all enzymes in QC-High, QC-Mid, and QC-Low materials was nearly identical in lot 1708 (10%) and lot 1808 (11%).

### 3.2. Categorical Results

Enzyme activities from the four levels of QC materials in both lots collectively straddled the three risk categories for all four enzymes tested (Table 2). Enzyme activities from both lots of the QC-BP material fell into the high-risk category for all four enzymes, and activities from both lots of the QC-High fell into the typical-range category for all four enzymes. Categorical results from the QC-Low and QC-Mid varied with respect to the enzyme. For GAA and GBA, all QC-Low results in both lots were in the high-risk category. For GLA, QC-Low results on both lots were mostly in the high-risk category, with a minority in borderline. For IDUA, QC-Low results on lot 1708 were mostly high-risk with the remainder borderline, while on lot 1808 the majority were borderline with a minority in high-risk.

## 4. Discussion

Newborn bloodspot screening for lysosomal storage disorders (NBS-LSD) has become increasingly widespread since it was first introduced using fluorescence-based assays [10] and further developed using MS/MS assays [11]. In the United States, Pompe disease, mucopolysaccharidosis I, mucopolysacchardosis II, and Krabbe have been added to the federal Recommended Uniform Screening Panel (RUSP), and more programs globally are including one or more LSDs in their screening panels. The CDC NSQAP has made DBS QC materials for LSD assays available since 2010, validating their performance in assays using mass spectrometry to measure activities for six lysosomal enzymes [7]. The availability of DMF assays for NBS-LSD prompted a comparable evaluation of these materials using the DMF method. CDC collaborated with MSPHL to assess the utility of these materials in a public health laboratory setting. MSPHL has extensive experience using DMF for NBS-LSD [8] and has established the optimal cutoff values for identifying newborns that may be affected by LSD and require follow-up testing. Starting with a pilot study in 2013, MSPHL has screened over 854,166 newborns for these four LSDs and detected 328 affected babies (infantile and late onset), with no missed cases reported. The cutoff values are adjusted periodically based on seasonal changes in the population distributions of enzyme activities.

Our results show that the DBS QC materials originally designed for validating NBS-LSD by the MS/MS method [7] were also suitable for validating the DMF assays, even though the activity levels between methods for the same enzymes differ significantly [9]. In designing these materials, we expected QC-BP to fall into LSD-affected ranges for all enzymes, and QC-High to fall into typical ranges for all enzymes. The 50% (QC-Mid) and 5% (QC-Low) were expected to encompass the lower-normal, borderline, and deficient ranges, depending on the particular enzyme. These latter controls would provide analytical QC materials in the critical ranges where cutoff values are assigned [12].

The results of this study, which used the MSPHL cutoff values to interpret the certification values obtained at CDC, confirmed the design intent of these DBS QC materials and their relevance in identifying LSD-affected newborns. The four levels of QC materials in each of the two DBS QC lots collectively spanned the categorical range from high-risk to borderline to no follow-up required for all four enzymes included in the DMF panel. For GAA, all replicates of each material in both lots fell into a single range, with no borderline results observed. For GLA, results in both lots from the QC-Low were mostly high-risk, with a few results in the borderline category. For GBA, only the QC-Mid results in both QC lots spanned more than one category, with the majority in borderline and a few in the typical range. For IDUA, only the QC-Low results spanned more than one category: lot 1708, having lower overall activity levels for QC-Low showed a majority in high-risk, a few in borderline, and none in the reference range (16/4/0), while lot 1808, having higher overall activity levels, showed the majority in borderline, several in high-risk, and one in the reference range (8/11/1).

The same QC materials used in these analyses were also analyzed at CDC by a method using tandem mass spectrometry (MS/MS). The activity levels measured by the two different platforms were markedly different, as shown recently by Dorley et al. [9]. GLA was the highest of the four enzymes as measured by both methods. The GLA activity differed between lots due to differences in innate residual activity and/or age from the time of collection of the core blood units used to create the QC materials. The remaining three enzymes differed in rank values, with GBA showing the lowest activity according to DMF, and IDUA showing the lowest activity according to MS/MS. The absence of an independent accuracy base for these enzyme activity assays underscores the need for QC materials that can monitor analytical consistency within different methods.

## 5. Conclusions

This study demonstrates that the CDC QC-DBS materials originally produced for NBS LSD MS/MS assays are also effective in validating and monitoring performance of enzyme activity assays measured by DMF, which can currently measure the following enzymes: acid α-galactosidase (GLA); acid β-glucocerebrosidase (GBA); acid α-glucosidase (GAA); and iduronidase (IDUA). By testing the CDC QC-DBS materials at regular intervals along with the QC material included in the DMF analytical system, NBS laboratories can better assess and document the reliability of results from their newborn DBS specimens.

## Figures and Tables

**Table 1:** Lysosomal enzyme activity mean and standard deviation results in µmol/hr/L blood on two lots of DBS QC materials analyzed by digital microfluidic fluorometry at CDC.

		Enzyme Activity Level in µmol/h/L Blood			
QC Level	QC Lot	GAA (1708/1808)	GLA (1708/1808)	GBA (1708/1808)	IDUA (1708/1808)
High	Mean	28.3/28.2	46.1/64.3	10.4/11.7	28.3/30.5
	SD	2.06/1.69	3.03/3.50	0.92/1.21	2.75/2.85
Mid	Mean	16.3/15.4	25.1/32.4	6.60/6.61	14.8/15.8
	SD	1.96/1.68	1.92/2.06	0.63/0.53	1.25/1.16
Low	Mean	2.52/2.43	5.22/5.59	2.28/2.36	3.30/4.23
	SD	0.22/0.33	0.49/0.61	0.20/0.41	0.63/1.03
Base Pool	Mean	0.97/1.44	3.74/3.66	2.05/2.17	1.52/2.44
	SD	0.24/0.70	0.44/0.39	0.26/0.20	0.73/0.67

GAA: acid α-glucosidase; GLA: acid α-galactosidase; GBA: acid β-glucocerebrosidase; IDUA: iduronidase.

**Table 2:** Categorical results for lysosomal enzyme activities measured by digital microfluidic fluorometry on two lots of DBS QC materials produced by and analyzed at CDC.

Summary Categorical Results on 1708 and 1808 QC Materials from CDC Analyses					
		High-Risk (Referral)/Borderline (Repeat)/Within Normal Range			
QC Level	Lot	GAA	GLA	GBA	IDUA
Base Pool	1708	20/0/0	20/0/0	20/0/0	20/0/0
	1808	20/0/0	20/0/0	20/0/0	20/0/0
QC-Low	1708	20/0/0	18/2/0	20/0/0	16/4/0
	1808	20/0/0	15/5/0	20/0/0	8/11/1
QC-Mid	1708	0/0/20	0/0/20	0/15/5	0/0/20
	1808	0/0/20	0/0/20	0/16/4	0/0/20
QC-High	1708	0/0/20	0/0/20	0/0/20	0/0/20
	1808	0/0/20	0/0/20	0/0/20	0/0/20

GAA: acid α-glucosidase; GLA: acid α-galactosidase; GBA: acid β-glucocerebrosidase; IDUA: iduronidase; BP: base pool.

## Data Availability

The data presented in this study are available on request from the corresponding author.

## Abbreviations

BP, Base Pool; CDC, Centers for Disease Control and Prevention; DBS, Dried Blood Spot; DBS QC, Dried Blood Spot Quality Control Material; DMF, Digital Microfluidics Fluorometry; LSD, Lysosomal Storage Disorder; MSPHL, Missouri State Public Health Laboratory; NBS, Newborn Bloodspot Screening; NSQAP, Newborn Screening Quality Assurance Program; RUSP, Recommended Uniform Screening Panel; UCB, Umbilical Cord Blood.
